# Factors affecting heat resilience of drone honey bees (*Apis mellifera*) and their sperm

**DOI:** 10.1371/journal.pone.0317672

**Published:** 2025-02-07

**Authors:** Alison McAfee, Bradley N. Metz, Patrick Connor, Keana Du, Christopher W. Allen, Luis A. Frausto, Mark P. Swenson, Kylah S. Phillips, Madison Julien, Zoe Rempel, Robert W. Currie, Boris Baer, David R. Tarpy, Leonard J. Foster

**Affiliations:** 1 Department of Biochemistry and Molecular Biology, Michael Smith Laboratories, University of British Columbia, Vancouver, British Columbia, Canada; 2 Department of Applied Ecology, North Carolina State University, Raleigh, North Carolina, United States of America; 3 Center for Integrative Bee Research (CIBER), Department of Entomology, University of California Riverside, Riverside, California, United States of America; 4 Department of Microbiology & Plant Pathology, University of California Riverside, Riverside, California, United States of America; 5 Department of Molecular, Cell & Systems Biology, University of California Riverside, Riverside, California, United States of America; 6 Department of Entomology, University of Manitoba, Winnipeg, Manitoba, Canada; University of Carthage, TUNISIA

## Abstract

Extreme temperatures associated with climate change are expected to impact the physiology and fertility of a variety of insects, including honey bees. Most previous work on this topic has focused on female honey bees (workers and queens), and comparatively little research has investigated how heat exposure affects males (drones). To address this gap, we tested body mass, viral infections, and population origin as predictors of drone survival and sperm viability in a series of heat challenge assays. We found that individual body mass was highly influential, with heavier drones being more likely to survive a heat challenge (4 h at 42°C) than smaller drones. In a separate experiment, we compared the survival of Northern California and Southern California drones in response to the same heat challenge (4 h at 42°C), and found that Southern Californian drones ― which are enriched for African ancestry ― were more likely to survive a heat challenge than drones originating from Northern California. To avoid survivor bias, we conducted sperm heat challenges using *in vitro* assays and found remarkable variation in sperm heat resilience among drones sourced from different commercial beekeeping operations, with some exhibiting no reduction in sperm viability after heat challenge and others exhibiting a 75% reduction in sperm viability. Further investigating potential causal factors for such variation, we found no association between drone mass and viability of sperm in *in vitro* sperm heat challenge assays, but virus inoculation (with Israeli acute paralysis virus) exacerbated the negative effect of heat on sperm viability. These experiments establish a vital framework for understanding the importance of population origin and comorbidities for drone heat sensitivity.

## Introduction

Heatwaves are expected to increase in frequency and severity as the climate changes [1], threatening the fertility of many animals, including insects [2]. Extreme temperatures within the range of what may occur during summer heatwaves (35–45°C) can negatively impact the fertility of flour beetles (*Tribolium castaneum*) [3], fruit flies (*Drosophila sp*.) [4], bumble bees (*Bombus* spp.) [5, 6], fruit moths (*Grapholita molesta*) [7], parasitoid wasps (*Anisopteromalus calandrae* and *Aphidius avenae*) [8, 9], and honey bees (*Apis mellifera*) [10–12], among others [2].

Although many insect species are affected by heat stress, the potential impact on honey bees is particularly concerning because they are the most widely used pollinating species in agricultural systems [13, 14]. Honey bees tolerate a wide range of climates, with native populations in Africa, Asia, and Europe; however, queens (reproductive females) and drones (reproductive males) are susceptible to heat-induced loss of fecundity, with significant death of spermatozoa resulting from heat exposure [10–12, 15]. Queens can survive heat-stress, but the viability of sperm cells stored within their spermathecae diminishes when queens are exposed to temperatures above 38 ˚C for an excess of 2 hours (h) [10]. Similarly, spermatozoa within drones also perish after heat exposure [11]. These losses of sperm viability are permanent reductions in fertility, since queens only mate during a short period early in life and cannot acquire more sperm later, while drones only produce sperm during development and not adulthood [16]. Concerningly, drones, despite having similar body masses [16], are more susceptible to heat-induced mortality than queens [10, 11].

While it is well known that honey bee colonies can maintain remarkably stable temperatures in the brood nest [17], little data exist on thermal fluctuations inside colonies during heatwaves. However, one study conducted in California reported that temperatures exceeding 42 ˚C can occur inside colonies during a heatwave (when ambient temperatures reached 45°C and access to water is limited), with especially hot temperatures at the nest periphery [10] where sexually mature drones tend to congregate [18]. Additional studies, where temperatures in the brood nest reached up to 40°C, corroborate that dangerously hot temperatures can occur inside colonies [19, 20]. Although yet unresearched (to the best of our knowledge), it is also possible that drones could experience extreme temperatures during bearding behavior, when large numbers of bees exit the nest under extreme heat conditions to minimize internal heat production [21, 22]. Therefore, despite the ability of colonies to thermoregulate under heat exposure to some extent [22–24], drones are likely still at risk of heat stress under extreme conditions, which are likely to become increasingly common [25, 26]. Although they are clearly indispensable for reproduction, little is known about the factors governing how heat impacts drone survival and fertility.

Two previous studies have shown that drones are sensitive to heat-induced mortality, but they do not agree on the magnitude of this sensitivity. McAfee et al. [10] reported that 50% of drones survived a 6 h treatment at 42 ˚C, whereas Stürup et al. [11] reported that just 23% of drones survived a shorter (4 h) treatment at the same temperature. While there are many potential explanations for discrepancies in results between laboratories, two possibilities ― in part motivating this research ― are that 1) drones derived from different populations could be adapted to different climates, and therefore exhibit different heat tolerance, and 2) variation in other characteristics, such as body mass or interactions with other stressors (*e*.*g*., pathogenic infections) may cause some drones to be more resilient to heat than others. One might expect heavier and larger drones to be more heat-resilient, a relationship that has sometimes [27–33], but not always (*e*.*g*., [29, 34, 35]), been found in other insect species. Since we have previously shown that drone body mass positively correlates with the number of viable sperm contained within the drone [36], suggesting greater reproductive investment, heavier drones may also be expected to produce higher-quality sperm that are more resilient to stress, such as heat. Conversely, pathogenic infections could be expected to have a positive or negative effect on heat resilience, depending on the pathogen in question. For example, pathogens could reduce individual fitness either through direct effects of disease or through a reproduction-immunity trade-off, as has been observed in other insects [37–39] (though a recent correlational analysis does not support this idea in honey bee drones [40]). Or, since there is evidence for cross-talk between antiviral immunity and heat stress response proteins [41], it is conceivable that some viral infections could counterintuitively improve heat tolerance (discussed further below).

Despite insects generally exhibiting relatively little variation in upper thermal resilience limits [42], heat resilience of terrestrial ectotherms does decline with increasing latitude [43] (*i*.*e*., along a gradient from tropical to temperate climates). Previous research shows that honey bees originating from Saudi Arabia (*Apis mellifera jemenitica*) are significantly more tolerant to heat than honey bees originating from Europe (*Apis mellifera carnica*) [44], which is consistent with such a latitudinal trend. Moreover, honey bee ancestry analyses conducted in Colombia show that colonies at low elevations (warmer temperatures) were enriched for African ancestry, whereas colonies at high elevations (cooler temperatures) were enriched for European ancestry [19, 45]. In addition, protein expression patterns in worker honey bees sampled across a latitudinal gradient show evidence of local climate adaptation [46]. Whether the patterns documented for workers extend to reproductive individuals and different populations has not yet been tested. Expanding our knowledge in this area may help predict the outcomes of different honey bee lineages or stocks in a changing climate.

A hotter climate may also lead to surprising interactions with diseases. There is growing evidence that heat-shock proteins (HSPs) ― a conserved group of proteins whose expression normally increases in response to heat ― are, though generally functioning to reduce protein misfolding and aggregation [47, 48], also an important part of the antiviral immune response in honey bees [41] and other insects [49]. This suggests that temperature exposure and viral infection may have interactive effects on honey bee physiology. McMenamin *et al*. [41] demonstrated that both heat exposure and viral inoculation elevated HSP expression, and exposing worker honey bees to heat shortly after infecting them with a virus improved the bees’ ability to clear the infection. It is thus conceivable that the reverse could be true ― that prior virus infection could improve bees’ ability to tolerate heat, in terms of either drone or sperm survival, through the dual roles of HSPs. Neither scenario has been investigated in drones, despite drones being disproportionately affected by viruses due to preferential parasitism by the *Varroa destructor* mite (a major vector of honey bee viruses) [50]. Astoundingly little is known about how viral infections affect drone fertility, and heat-by-virus interactions on drone fertility have not been explored.

Long-term data show that the number of heatwave days has increased the most in tropical regions [25]; however, extreme conditions can still occur in temperate climates, which are warming at a faster rate. During the heat event in Western Canada and the Pacific Northwest spanning June 25 to July 1, 2021 [51], temperatures were sufficiently extreme (up to 49.6°C) for beekeepers in the region to observe negative impacts on their colonies, including colony death [52]. Therefore, beekeepers in high-latitude, temperate regions will likely need to begin employing heat-management strategies that have not been historically necessary. Such strategies may include mechanical approaches (*e*.*g*., year-round hive insulation, shading, *etc*.), which could support colonies over shorter periods of heat exposure. Should beekeeper management of hive temperature become ineffective or impractical, strategic stock selection and breeding for climate resilience will be a crucial component of an integrated management plan. Such an endeavor will rely on first identifying relationships between heat resilience and population origin, among other factors, in existing stocks.

Southern California was not seriously impacted by the 2021 heat wave described above, but this region has also seen a shift toward more extreme temperatures in general. The geographic area has historically been classified as semi-arid (Köppen climate classification BSk), but recently it has become more similar to an arid climate (Köppen climate classification BWh) with hot, dry summers. Concerningly, the frequency of heatwaves have rapidly increased in Southern California since 1950 [53]. For example, a major heatwave from September 5–7, 2020, in Riverside County caused daytime highs to reach 47°C [54]. Interestingly, genetic analysis of unmanaged (feral) honey bees in Southern California shows that they possess approximately 38% African ancestry [55]. Since African honey bees survive in hot climates, and unmanaged honey bees in Southern California have been historically and increasingly exposed to intense heat, we expect bees from this population to possess adaptations to heat stress.

In this work, we investigated factors affecting heat resilience of honey bee drones and their sperm. We tested the influence of population origin and body mass on drone and sperm survival during heat challenge assays, as well as the impact of adult-acquired viral infection on sperm heat resilience. First, to investigate the relationship between body mass and survival probability, we recorded adult body mass and survival after four-hour heat challenges at 42°C. In a second heat challenge experiment, we assessed potential local adaptation by comparing drone survival between Southern Californian and Northern Californian lineages, with Southern California characterized by an arid climate with more extreme maximum temperatures than Northern California. Finally, we investigated relationships among body mass, virus inoculation, and heat exposure using *in vitro* heat challenge assays in which sperm was extracted from drones and subjected to an array of temperatures and durations. We hypothesized that 1) heat resilience of drones and their sperm would reflect the local climates of their geographic population origin (*e*.*g*., drones from Southern California would be more heat resilient than drones from Northern California), 2) that heavier drones would be more likely to survive a heat challenge than lighter drones and would produce sperm with greater heat resilience, and 3) that due to crosstalk between heat-shock and antiviral gene expression, viral infection would positively influence heat resilience of sperm.

## Methods

Experiments were conducted at three different locations: the University of British Columbia (UBC), University of California Riverside (UCR), and North Carolina State University (NCSU). Because experiments at different institutions used different methodologies, such as testing drones of different ages, results of individual experiments are not always directly comparable to one another and are best assessed as independent tests assessing relative responses. In addition, there are some differences in heating methods, depending on the requirements of the particular experiment. For example, for the experiments conducted at UBC that evaluate *in vitro* sperm survival (Experiment 2), we aimed to heat the sperm cells as similarly as possible to the drone heating method (*i*.*e*., heating in the same incubator that the drones were heated in). For the *in vitro* sperm viability time course experiment (Experiments 4), better heat conduction was necessary as the sperm required fast heating at multiple temperatures simultaneously; therefore, a thermocycler was used.

### Animal ethics

As non-cephalopod invertebrates, honey bees are not subject to animal ethics committee approval at the participating institutions.

### Colony management at UBC

On March 31^st^, 2022, we determined *Varroa* mite levels of overwintered colonies at UBC using the alcohol wash method [56]. On April 6^th^, 2022, we subsequently treated any colonies yielding > 0.5 mites per 100 bees with formic acid (Formic Pro, NOD Apiary Products, Ltd.), according to the manufacturer’s instructions, to ensure minimal interference of mites with drone brood rearing. Stock was composed of a mix of overwintered (2021) queens from Northern California as well as new (2022) queens from Ukraine and Australia.

We fed all colonies 50% sugar syrup and 15% pollen patties (Global) on a weekly schedule to encourage population growth and initiate drone rearing. In May 2022, we confined established queens on drawn drone comb for three days using a single-frame excluder, which restricts the queen’s movement to a single frame but allows workers to pass freely. Any eggs the queen laid developed *in situ*. Drone development takes 24 days on average [16] and the queen normally requires approximately 1–2 days to lay a frame of comb; therefore, twenty-six days after the queens were initially caged on drone comb, we returned to the colonies and marked callow or actively emerging drones with a Posca paint pen. Callow (≤ 1 day old) drones are easily recognizable by their soft bodies and light grey appearance.

To maximize recapture rates, six days after paint-marking drones (the minimum time for sperm maturation to occur [36], but when most drones are not yet flying), we returned to the colonies and collected marked drones into cages made of inverted, clear plastic 12 oz (355 mL) cups. The cages had ~100 air holes (1 mm diameter) melted in the sides with a hot metal comb, and the floor of the cage was made of a wire mesh disc that was hot glued on to the rim. Between 20 and 50 drones were held in each cage (two cages per colony), depending on how many could be recaptured from each colony, as well as an equal number of attendant workers as drones, which were collected from the same frames as the drones. These attendant workers are necessary for adequate drone care. Caged bees were held overnight in a 33°C incubator (typical hive temperature, which ranges from 33–35°C in the brood nest, with more variable temperatures toward the periphery [16]) with access to 50% sugar syrup fed by a dental wick pressed against the mesh floor as well as a 2 mL microfuge tube packed with Ambrosia fondant in the cage (Nordzucker, Germany). Since callow drones were already between zero and one day old at the time of marking, the drones were seven to eight days old at the time of heat challenge (below).

### Experiment 1 –Relationship between adult drone mass and heat survival

To conduct experiment 1, the next day, we moved half the cages (one for each colony, N = 6 colonies) to the hot incubator (heat challenge group; 42°C, 60% relative humidity, N = 278 drones in total), while the other cages remained in the 33°C incubator (control group, N = 220 drones) (see **S1 Table in S1 File** for complete sample size information per colony). After four hours, the number of dead (immobile) and live (mobile) drones in all cages were recorded. This is a realistic time frame during which drones might experience extreme heat in the field, such as when congregating at the nest periphery during the hottest part of the day [10]. Dead drones from the heat challenge group were removed with forceps and weighed. Next, the remaining bees were anaesthetized with carbon dioxide (10 min), workers were removed, and the live drones were weighed. All drones were then frozen in 50 mL tubes for later processing.

### Experiment 2 –Relationship between sperm viability and heat exposure

For experiment 2, ten to twelve drones from each UBC colony (N = 10 colonies) were used for an *in vitro* sperm viability challenge, which enabled the response of sperm viability to heat challenges to be assessed without interference of survivor bias, and for the sperm sample to be split into paired heat and control exposures (N = 136; see **S2 Table in S1 File** for group-wise sample size information). We briefly anaesthetized the drones with carbon dioxide (1 min), then dissected out their seminal vesicles, which we gently ruptured in 100 μl of Buffer D (17 mM D-glucose, 54 mM KCl, 25 mM NaHCO_3_, 83 mM Na_3_C_6_H_5_O_7_) [57] with a plastic pestle. For some colonies, we obtained sperm viability data but no drone survival data, which occurred when we recaptured sufficient drones for viability testing but not the larger number needed for survival.

We diluted 10 μl of the sperm solution in 90 μl Buffer D in two separate tubes (one tube for the heat challenge and one control tube). Once all sperm samples were prepared (100 μl per tube), half the tubes were moved to the same incubator used for the heat survival challenge (42°C) and half the tubes were moved to the control incubator (33°C). After four hours, we assessed sperm viability by dual fluorescent staining using SYBR 14 and propidium iodide (Invitrogen™ LIVE/DEAD™ viability/cytotoxicity kit) for both heat-challenged and control samples as previously described [57]. Sperm images were taken using a Cellomics HCS (Thermo) microscope at 200x magnification. Once all images were acquired, a colleague not otherwise involved in the study renamed all files with a random character tag so that the stained sperm cells were counted blindly.

In addition to conducting sperm viability heat challenges using drones from UBC colonies, we also conducted sperm viability heat challenges using drones supplied by beekeepers throughout British Columbia. July 2022, we received drones from six queen producers located in the following regions: Sunshine Coast (Source A), Fraser Valley (Source B), North, Central, and South Okanagan (Sources C, D, and E, respectively), and Nechako (Source F) regions (see **S2 Table in S1 File** for complete sample size information). The donors each sampled 7–10 sexually mature drones (N = 55 total) using the “buzz test” (selecting drones that buzzed strongly when pressed on the thorax against the comb as an approximation for sexual maturity) [58]. The drones were packed in plastic queen shipping cages (JZ-BZ) with fondant and one drone per cage with five attendant workers. The cages were then shipped in plastic JZ-BZ battery boxes by express courier (1–2 day shipping), with loose nurse bees and digital temperature loggers; an approach that yielded 100% survival with no extreme temperatures (< 15°C or > 38°C) observed. All drones were processed two days after being shipped by the donor, even if they arrived after one day, to keep the caging time constant. We conducted the dissections and sperm viability measurements as described above. Samples were excluded if fewer than 75 sperm cells could be counted across three 200x images or if sperm aggregation impeded the accuracy of counts.

### Southern Californian and Northern Californian queen sources and colony management at UCR

In April and May 2023, Northern California queens were sourced from packages and nucleus colonies. Southern California queens were acquired from two different sources: 1) donated by swarm removal specialists in Riverside, Orange, and San Diego counties; and 2) colonies originally sourced from Northern California which have requeened and open mated with local Southern Californian drones for at least three generations. We have confirmed that the drones from Northern California and Southern California used in this study are two distinct populations (Allen et al., unpublished). All colonies were fed *ad libitum* with pollen and sugar syrup. Oxalic acid vapor treatments for *Varroa* mites were used when colonies reached thresholds above 3% (determined using alcohol washes [56]). Treatments were not applied during the drone rearing period.

By June 2023, we replaced a single frame in all colonies (N = 3 Northern Californian, N = 5 Southern Californian) with drawn drone comb. Colonies were checked weekly to observe egg laying and drone development. Callow drones were marked with a Posca paint pen (Uni Mitsubishi Pencil, Japan) and different colors were used to associate drones to specific marking dates, since the number of colonies tested was too great to conduct simultaneously. After aging for 14 days, drones were collected and placed into 3D-printed plastic holding cages (10 x 6 x 12 cm) with 30 worker bees. Water and honey were fed *ad libitum* before and during the heat treatment.

### Experiment 3 –Northern Californian vs. Southern Californian drone heat survival

For experiment 3, between 20 and 30 drones were placed into each cage (1 cage per colony for control and ~3 cages per colony for heat treatment, depending on availability of drones in the colony). Tests conducted at UBC showed that drone survival at control temperatures was very high (close to 100%); therefore, fewer drones were included in the control group than the heat treatment group in order to maximize our ability to see differences in heat resilience between population origins. A total of N = 429 drones from Northern Californian colonies and N = 513 from Southern Californian colonies were tested; see **S3 Table in S1 File** for complete sampling information. After collection, cages were immediately placed into control (35°C) and heated (42°C) incubators for 4 hours at 60% relative humidity. The number of live and dead drones were counted in each cage at the end of the treatment.

### Queen sources and colony management at NCSU

Honey bee colonies of mixed European origin, headed by a mix of locally-reared queens and commercial queens purchased from California, Hawaii, or Georgia were maintained at the Dix Honey Bee Research Facility in Raleigh, North Carolina and tested on campus at NC State University. Drones sourced from these colonies were used to conduct two sperm viability temperature-by-time arrays: experiment 4 (a temperature-by-time array for drones from 6 different colonies and natural variation in viral infections and body mass) and experiment 5 (a time-series experiment using virus-inoculated drones).

### Experiment 4 –Effects of background virus infections on sperm viability in heat challenges

For experiment 4, a single round of sampling was performed in June 2021 (32 drones from 5 colonies; see **S4 Table in S1 File** for complete sample size information). Sexually mature drones were collected while returning to the nest entrance after flying (typically > 7 d old [59]). The drones were placed into 15 x 15 cm cages of wood and wire mesh and stored briefly in a separate colony until experimental use. The drones were weighed, manually ejaculated [60], then semen was collected into a glass syringe using a Harbo extraction needle [61]. Semen was placed into a 0.25 mL plastic strip tube containing 100 μl buffer D [36] and lightly vortexed. 10 μl was immediately removed and diluted 1:10 into buffer containing dyes from the Invitrogen Live/Dead Sperm viability kit (Thermo-Fisher Scientific) to assess baseline (time = 0) sperm count and viability using a Nexcelom Cellometer (Nexcelom Bioscience, LLC). The remaining sperm sample was then aliquoted into strip tubes, placed into a thermocycler, and sperm samples from eight drones each (not pooled) were held at 30 ˚C, 45 ˚C, 52.5 ˚C, and 60 ˚C. Sperm was repeatedly sampled at 0, 35, 65, 125, and 245 minutes to measure sperm viability. Drone carcasses were then processed via RT-qPCR to determine absolute copy numbers of naturally occurring, common honey bee viruses (acute bee paralysis virus (ABPV), black queen cell virus (BQCV), chronic bee paralysis virus (CBPV), deformed-wing virus A (DWV-A), deformed-wing virus B (DWV-B), Israeli acute paralysis virus (IAPV), and Lake Sinai virus (LSV)) following methods exactly as described by Lee *et al*. [62]. This enabled us to investigate viral infections as covariates potentially explaining differences in baseline sperm viability. See Lee *et al*. [62] for information on primers, RNA extraction, cDNA synthesis, and all other methods relevant to qPCR analysis.

### IAPV isolation and propagation

The Israeli acute bee paralysis virus (IAPV) inoculum used for further propagation was sourced from existing stock material that had been stored at -80°C. The virus was propagated and repurified as described in Chapman *et al*. [63]. After passing the IAPV extracts through a Corning syringe filter (0.45 μm) to remove bacteria, 1.0 μl of virus solution was injected into pupae between the 2nd and 3rd tergites using a Hamilton syringe with a 30GxK gauge sterile needle. White to pink-eyed pupae that contained low background virus levels (as determined by PCR) were obtained from colonies that had been initiated from Australian bee packages prior to the introduction of varroa in that country (Aussie bees® sourced from Tasmania). These bees were hived on new plastic foundation and held in isolated apiaries in Canada (Star Lake, Manitoba, 49° 45′ 08″ N 95° 15′ 24″ W and Red Deer Lake, Ontario 50° 00′ 47″ N 94° 11′ 26″ W), in areas where no other managed honey bees occur. The continued absence of *Varroa* mites in the colonies was confirmed by evaluating sticky boards and alcohol washes [56]. Inoculated pupae were place in a covered Petri dish with slightly damp filter paper and incubated at 31°C. Any pupae that reached the adult stage prior to the end of 5 days were either homogenized immediately after collection or were stored at −80°C for later processing.

### IAPV purification

For virus purification, the pupae were placed in 50 mL Spex SamplePrep polycarbonate cryovials with two 9 mm steel beads and homogenized at 1750 rpm for 3 min in a Spex SamplePrep Geno/Grinder® 2010. To make primary extracts, 2 mL of extraction buffer (0.01 M buffer phosphate, pH 7.2, containing 0.02% sodium diethyldithiocarbamate trihydrate (DETCA), Sigma-Aldrich Cat #228680) and 0.5 mL chloroform were added per 1 g of homogenate and samples were kept on ice in a fume hood. Each sample was vortexed for 30 s, then centrifuged at 8000 *g* (8,700 rpm, Eppendorf Centrifuge 5430, placed in the fridge) for 10 min at 15°C. From each tube, the supernatant was collected into a 5 mL centrifuge tube based on sample origin. Samples were stored at −80°C until further purification. High speed centrifugation was done to further purify the virus samples. The primary extracts were thawed and added to 35 mL Thermo Scientific™ PA Ultracrimp Tubes, with the rest of the volume filled with more extraction buffer. The tubes were centrifuged at 15°C for 3 h, at 75,000 *g* (27,200 rpm, Sorval Discovery 100SE, Sorval T-865 Rotor). The supernatants were discarded, and the pellets were placed in new 5 mL centrifuge tubes. The pellet was resuspended in 1 mL of 0.5 M potassium phosphate buffer (pH 7.2), 2% Brig-58 (Sigma-Aldrich Cat#P5884) and 0.2% sodium deoxycholate (Sigma-Aldrich Cat#D6750).

The solutions were mixed using a spatula and by vortexing. The samples were left overnight at 4°C and then mixed gently again in the morning. The samples were split into 2 mL microcentrifuge tubes to be centrifuged for 15 min at 4°C, at 8000 *g* (8700 rpm, Eppendorf Centrifuge 5430, placed in the fridge). The supernatants were then pooled into a new 5 mL centrifuge tube. The samples were then passed through a 0.45 μm syringe filter into new 5 mL centrifuge tubes, and 20 μL of each sample was taken for PCR analysis to determine the final concentrations and purities. The final extract was 99.69% pure IAPV (with other components consisting of 0% DWV-A, 0.31% DWV-B, 0% BQCV, and 0% SBV).

### Experiment 5 –Effect of newly acquired virus infection on sperm viability in heat challenges

For this experiment, two rounds of sampling were performed, the first terminating in June 2023 and the second terminating in July 2023 (see **S5 Table in S1 File** for complete sampling information). For each round, drones from a single colony were collected from the nest periphery. Drones were collected according to the “buzz test” as previously described [58] and caged as in experiment 4; for each round drones from a single colony were collected. Tube feeders (5 mL) were stocked with 50% sucrose and tap water. Cages were also supplied with a solid feeder stocked with pollen patty made from ground pollen mixed with 50% sucrose, as well as 5 g wet honey cappings placed on the cage bottom, which we found to improve drone survival. Liquid food and water were replaced as needed. Each cage was stocked with 100 workers and 30 drones. Cages were kept in a dark incubator set to 30°C and ambient humidity. This temperature is lower than the temperature of the brood nest but still within drones’ thermal preferences [64].

Drones were either uninjected (control), injected with 1x PBS (sham-inoculated), or injected with 500 copies/μl IAPV (IAPV-inoculated) in 1x PBS. IAPV was used because the symptomatic nature of IAPV can be an asset for visually determining success of experimental infections. Injections were conducted with a microinjector with capillary glass needles (Tritech Research) calibrated to deliver approximately 1 μl per injection as assessed by loading 10 μl of 1x PBS and counting the number of injections until the full volume was delivered. A single calibrated needle was used for the whole study where possible, with sham injections preceding the virus injections in all cases to minimize risk of contamination. Needle calibration was checked after every new treatment and after each new needle installation. Drones were injected through the intersegmental membrane between the 5^th^ and 6^th^ abdominal segments, dorsal to the overlap between the tergite and sternite. Any drone that displayed signs of lethargy, injury, or ejaculation after injection was discarded. Drones were kept for three days after injection, in a dark incubator set to 30°C and 25% RH (ambient for the room), at which point the survivors were collected for subsequent analyses. Final surviving drone sample sizes were n = 9 drones in each experimental group (IAPV-inoculated, sham-inoculated, and uninjected).

To assess the sperm viability, drones were removed from the field facility and returned to the laboratory where they were prepared for dissection. Drones were dissected following previously described methods [36]. Briefly, live drones were weighed, then reproductive maturity was determined based on mucus gland and seminal vesicle color [64]. The seminal vesicles were homogenized by light mixing with forceps in 100 μl buffer D [57]. Diluted spermatozoa were mixed by briefly vortexing and 10 μl of the spermatozoa sample was immediately diluted 1:10 into buffer containing dyes from the Invitrogen Live/Dead Sperm viability kit (Thermo-Fisher Scientific) to assess baseline (time = 0) sperm count and viability as for experiment 4. The remaining sperm sample was then allocated into strip tubes and placed into a thermocycler held at 52.5°C and repeatedly sampled at 1, 2, and 4 hours. Drone carcasses were then processed via RT-qPCR to determine absolute copy numbers of IAPV as well as additional background viral targets as described for experiment 4.

### Statistical analysis

All statistical analyses were conducted in R (version 4.3.0) [65] using the packages nlme and lme4 [66, 67]. Appropriateness of fit was confirmed using tools within the DHARMa package [68] for all linear and generalized linear mixed models described below.

### Drone survival

To determine the impact of heat on drone survival (experiment 1), we used a generalized linear mixed model (glmer) with survival as an independent binomial response variable (1 = survived, 0 = died), treatment (levels = heat and control) as a predictor variable, and colony (six levels) as a random effect.

To determine if survival was linked to body mass among heat-treated drones, we used a generalized linear mixed model (glmer) with survival as a binomial response variable (1 = survived, 0 = died), body mass (continuous) and source (factor, 3 levels) as predictors, and colony as a random intercept term (6 levels). Only drones that were both exposed to heat and subsequently weighed were analyzed here (N = 257 individuals; Table 1).

**Table 1:** Post-hoc testing of sperm viability heat-control contrasts for BC drones.

Source	BC region	Estimate	Z ratio	P value*
A	Sunshine Coast	4.9	6.3	< 0.001
B	Fraser Valley	5.1	6.3	< 0.001
C	North Okanagan	1.5	2.7	0.20
D	Central Okanagan	2.7	2.8	0.18
E	South Okanagan	4.2	3.3	0.043
F	Nechako	1.3	0.81	1.0

*Tukey method of p-value adjustment

We analyzed Northern Californian and Southern Californian drone survival data using the same approach as described above, except that drone mass was not recorded in this experiment, so only treatment (heat or control) and population origin (Northern or Southern) were included as interacting predictors. Post-hoc testing was performed using emmeans [69] with the Tukey adjustment method.

### Sperm viability

To assess relationships between sperm viability, heat treatments, and population origin for the sperm viability challenge data, we again used generalized linear mixed models (glmer), with different transformations applied to the response variable (sperm viability percent) depending on the characteristics of each dataset. For the Australian, Ukrainian, and Northern Californian heat challenge sperm viability data, we observed that variance in sperm viability appeared to depend on heat treatment (variance in viability of heat-treated sperm was larger than control sperm). These sperm viability data were also non-normal, confirmed by a Shapiro test and density plots. To account for these two issues, we modeled proportion of dead sperm (rather than percent live sperm) using a gamma distribution. The gamma distribution is weighted heavily to smaller numbers and does not permit zeros (which are present in the live sperm parameter, hence using dead sperm instead), which is suitable for our data in this format. Our final model fit proportion of dead sperm against population origin (Australian, Ukrainian, and Northern Californian) and treatment (heat or control) as interacting terms, as well as drone as a random intercept variable to account for repeated measures (each drone’s sperm was split into control and heat treatments, and thus these samples are not independent).

To analyze the sperm viability heat challenge data of drones originating within British Columbia, we followed the same principles as above. We used a generalized linear mixed model (glmer) to model proportion of dead sperm against donor source (six levels; Sources A-F) and heat treatment (heat or control) as interactive explanatory variables, as well as drone as a random intercept variable. As above, we specified a gamma distribution. Post-hoc testing was conducted using emmeans [69] for pairwise contrasts (Tukey method).

The sperm viability data from the temperature-by-exposure time arrays (Experiments 4 and 5) had a different distribution compared to the other sperm viability datasets, and was best modelled by applying an arcsine square root transformation to sperm viability proportions. Data from Experiment 4 was then modelled using a linear mixed effects model (lmer) with transformed sperm viability as the independent variable and a three-way interaction term of temperature (numeric), exposure time (numeric), and body mass (numeric), since we predicted that the negative effects of higher temperatures and longer exposures would be moderated by heavier body masses of the original drones. Drone was included as a random intercept variable to account for differences in baseline sperm viability. Although 60°C was also tested as a heat treatment, we removed this group from the analysis as we were unable to design a well-fitting model when including it (this group yielded a disproportionately large number of outlier data points, determined by inspecting residual distributions and calculating Cook’s distance); however, we still display these data in the results. Post-hoc testing was not conducted for Experiment 4 data, as all predictors were continuous variables.

Experiment 5 data was also analyzed by applying an arcsine square root transformation to sperm viability. The linear mixed effects model included IAPV counts (ln-transformed) and exposure time (levels: 0, 1, 2, and 4 h) as interactive terms, treatment group (factor: levels = uninjected, sham-inoculated, and IAPV-inoculated) as a covariate, and drone as a random intercept. We would have preferred to treat exposure time as a continuous variable; however, preliminary tests showed that the approach yielded a poorly fitting model (quantile deviations were detected using tools within the DHARMa package [68]), whereas including time as a categorical variable did not. Both IAPV counts and treatment group were included in the model to account for variation in the IAPV infection intensity and to control for physical damage caused by injection. The ‘emtrends’ function within emmeans [69] was used to extract time point contrasts interacting with the IAPV counts predictor (*i*.*e*., time point contrasts for which the sperm viability-by-IAPV slopes are significantly different). In a separate analysis, differences in ln-transformed IAPV copy numbers between treatment groups were confirmed using a simple linear model (lm), with posthoc testing conducted using emmeans [69].

## Results

### Experiment 1 –Adult body mass predicts drone survival

**Fig 1:**
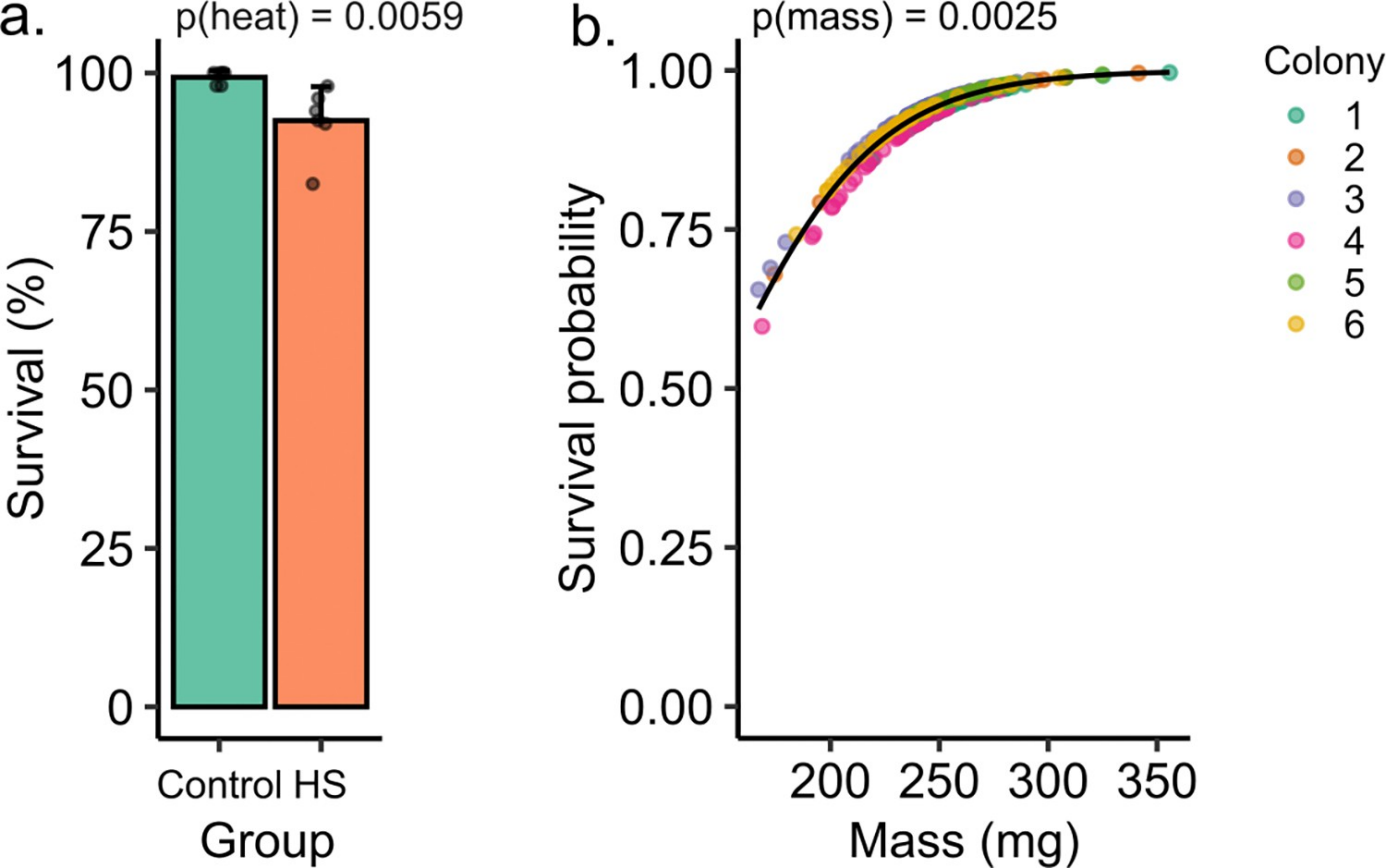
Honey bee drone survival in response to heat. We recorded wet body mass and survival of age-matched drones from six different colonies after a 4 h, 42°C heat challenge. A) Heat exposure (HS) significantly reduced drone survival (generalized linear mixed model). Dots represent average survival for each colony and error bars represent the standard deviation. B) Among the drones exposed to heat, body mass was a significant predictor of survival probability. Fitted survival probabilities were extracted and plotted against wet mass taken immediately after the heat challenge.

To determine if adult body mass influences heat resilience, we exposed age-matched drones from six colonies to a fixed temperature (42°C, 60% relative humidity), recorded their body masses, and compared their survival rates after 4 h. Survival rates were overall high in both the heat treatments (92.8%; N = 258 of 278) and control groups (99.1%; N = 218 of 220), but heat treatment did cause significant drone mortality overall (generalized linear mixed model, coefficient = -1.7, z = -2.8, p = 0.0059; **Fig 1A**). Among drones that were exposed to heat, we found that those with lower body masses were significantly more likely to have died during the heat exposure (coefficient = 0.028, z = 3.0, p = 0.0025; **Fig 1B**).

### Experiment 2 –Population origin predicts sperm heat resilience among British Columbian drones

**Fig 2:**
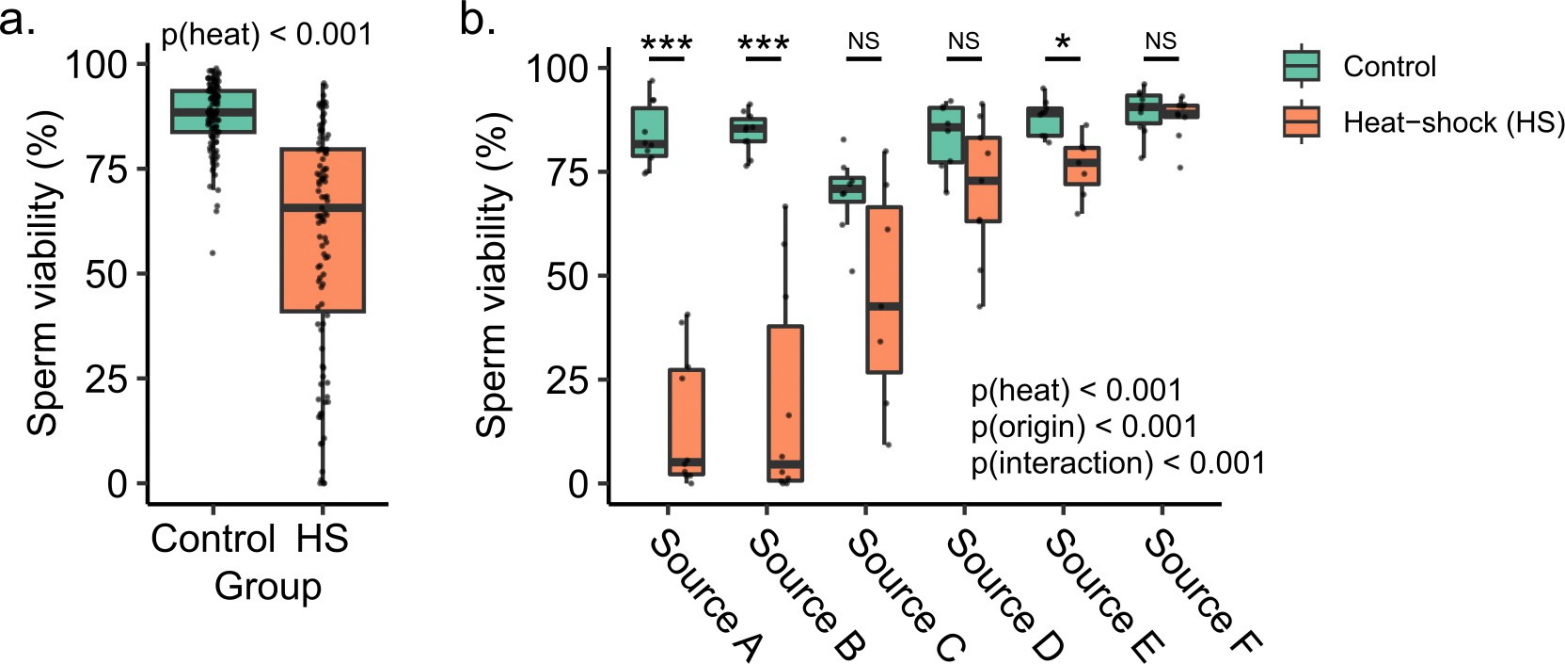
Population origin influences changes in sperm viability in response to heat. Sperm cells extracted from seminal vesicles were split into two samples and exposed to heat (HS) (42°C) or control (33°C) treatments for four hours, followed by viability analysis using dual fluorescent staining. Boxes represent the interquartile range, and whiskers span 1.5 times the interquartile range. The bar represents the median. Drone was included as a random intercept in generalized linear mixed models. A) Among UBC drones, heat had a strong negative but highly variable effect on sperm viability. B) Sperm viability from drones originating from six different population origins (sources) throughout British Columbia, Canada, varied according to a significant interactive effect between population origin and heat treatment. Source origins include the Sunshine Coast (Source A), Fraser Valley (Source B), North Okanagan (Source C), Central Okanagan (Source D), South Okanagan (Source E), and Nechako (Source F). NS = not significant; * p < 0.05; ** p < 0.01; *** p < 0.001. See **Table 1** for summary statistics.

To test if sperm from drones with different population origins responded to heat differently, we exposed extracted sperm cells to heat and compared sperm viability between treatments and origins. We first confirmed that our heat treatment methods do negatively impact sperm viability in our *in vitro* assays: Among drones from colonies maintained at UBC, heat significantly reduced sperm viability (**Fig 2A & S1 Fig in S1 File**; generalized linear model, coefficient = -0.051, t = -11.8, p < 0.001). Next, we used the same assay to assess impacts on viability of sperm from drones sourced from colonies propagated within British Columbia, Canada, at six different queen rearing operations (see Methods). Sperm viability was strongly affected by population source (χ^2^ = 92.0, df = 5, p < 0.001), heat (χ^2^ = 84.1, df = 1, p < 0.001), as well as an interaction between population origin and heat (χ^2^ = 22.8, df = 5, p = 0.00037; **Fig 2B**). Post-hoc testing shows that sperm from Source A, B, and E drones were significantly negatively impacted by heat while sperm from Source C, D, and F drones were not. Moreover, the magnitude of the heat effect was stronger for sperm from Sources A and B than Source E (see **Table 1** for statistical reporting).

### Experiment 3 –Southern Californian drones are more resilient to heat than Northern Californian drones

**Fig 3:**
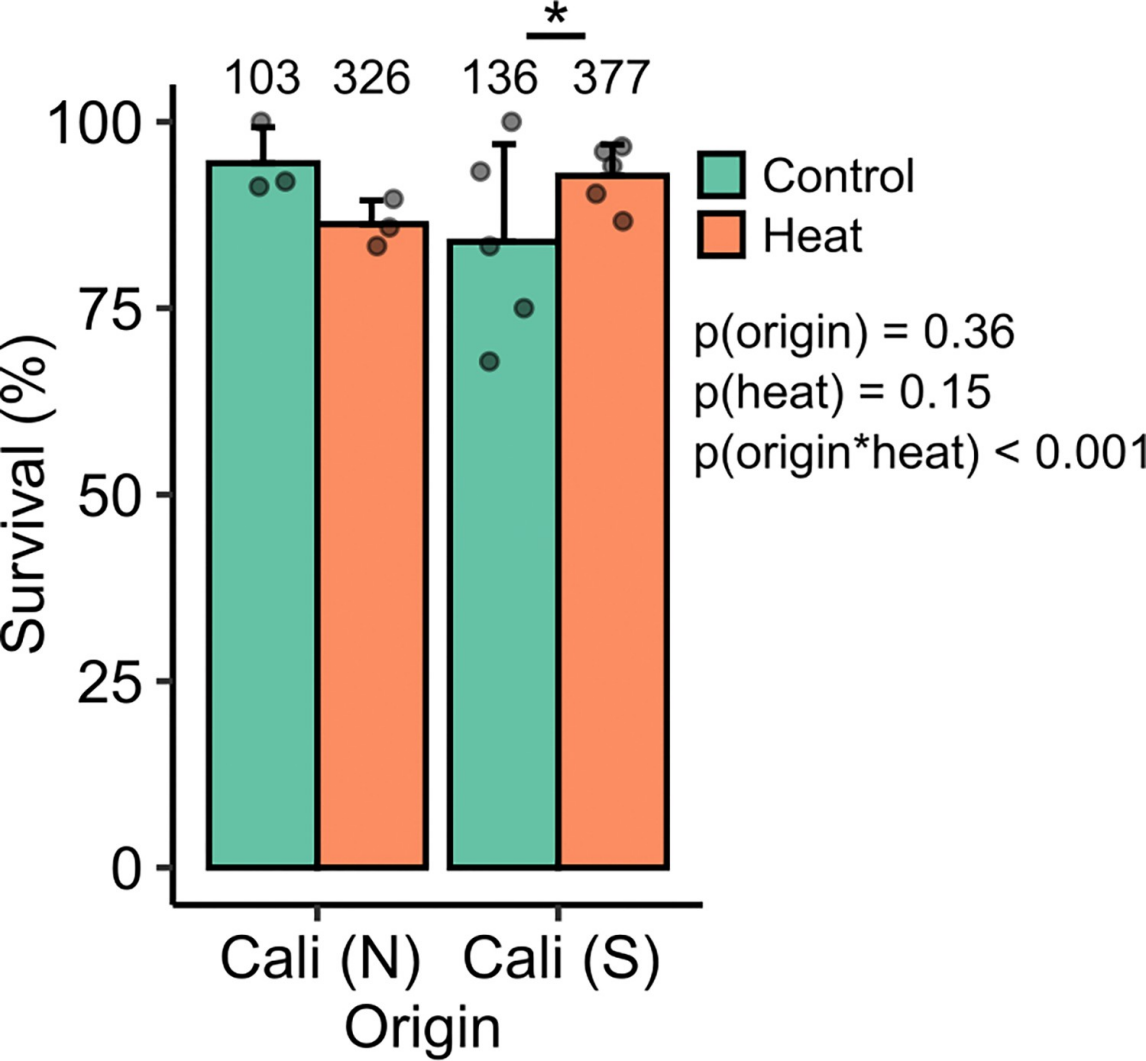
Heat challenge survival of Southern Californian and Northern Californian drones. Drone survival (1 = survived, 0 = died) was significantly predicted by an interactive effect between population origin (Northern and Southern Californian (Cali (N) and Cali (S), respectively) and heat treatment, as determined using a generalized linear mixed model (with origin and heat treatment as interacting factors, as well as a random effect of colony). The asterisk indicates the only significant pairwise contrast observed during post-hoc testing (general linear hypothesis test, Tukey method).

In an independent experiment, we used the same experimental design as used in experiment 1 to test survival of drones from Southern California compared to stock propagated in Northern California. We found a significant interaction between population origin and heat treatment (**Fig 3**; generalized linear model, χ^2^ = 12.8, df = 1, p < 0.001). Post-hoc testing of all possible contrasts shows that this interaction was driven by the high survival of heat-shocked Southern Californian drones relative to the control group, which was the only significant pairwise contrast (z = 3.2, p = 0.0067), whereas heat-shock tended to decrease survival Northern Californian drones (z = -2.1, p = 0.14). We unfortunately did not have the capacity to test changes in sperm viability in response to heat among drones from these populations at the time of these experiments, and this remains an important area of future study.

### Experiment 4 –Drone body mass is not linked to sperm heat resilience

**Fig 4:**
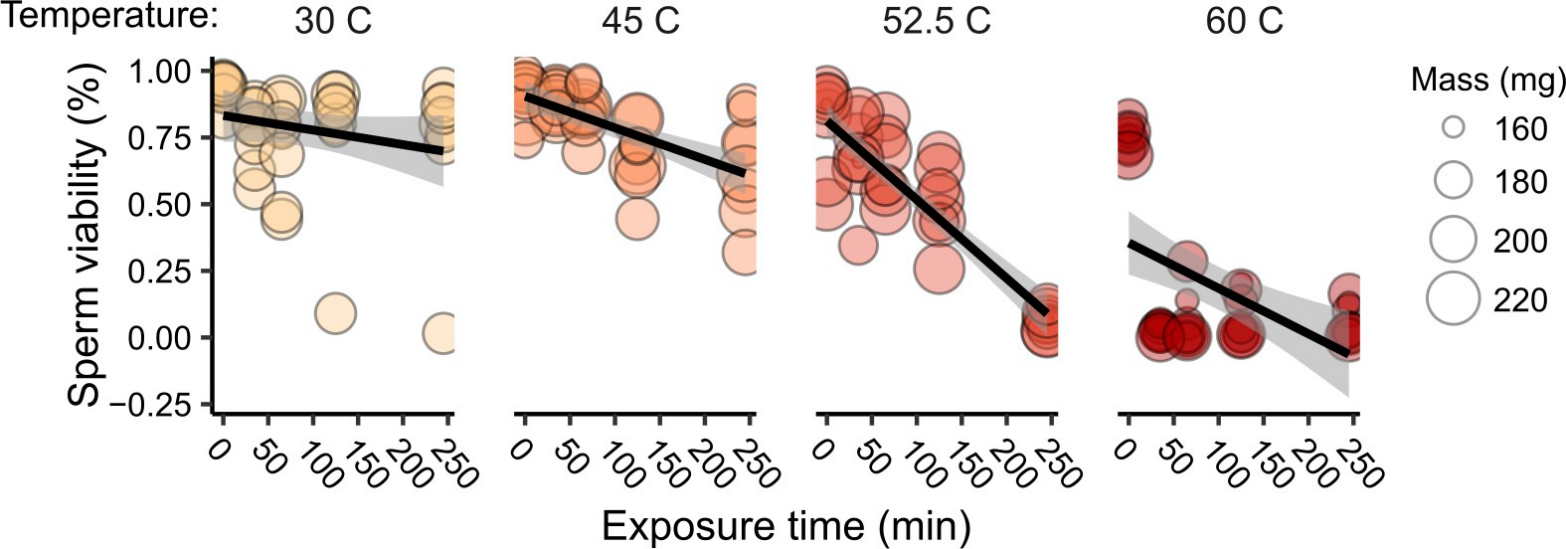
Interactions between temperature and time, but not body mass, predict sperm viability. We sampled sperm from 48 drones belonging to six colonies and challenged the sperm to an array of temperatures and exposure durations. We found significant main effects and interactive effects of heat and exposure duration on sperm viability (linear mixed model on arcsine square root-transformed viability proportions). Note that the 60°C temperature group is shown here but was not statistically modelled due to poor model fitting (see Methods). No post-hoc testing was conducted as all model predictors were continuous variables.

To test for potential explanatory factors underlying differences in sperm resilience to heat, we assessed relationships between body mass, underlying viral infections, and sperm heat resilience. We recorded wet body mass for N = 48 drones of mixed origins, tested them for a panel of seven honey bee viruses (see Methods), extracted their sperm, and subjected them to an *in vitro* array of temperatures and exposure durations to define the upper boundary within which sperm can maintain some viability. We found no three-way interaction between time, temperature, and body mass (one might expect sperm from heavier drones to take longer to be impacted by high temperatures); therefore, we removed body mass from the interactive term to increase power, but still did not identify a main effect of body mass on sperm viability (χ^2^ = 0.092, df = 1, p = 0.76). We did, however, identify significant effects of temperature (χ^2^ = 12.9, df = 1, p < 0.001), exposure time (χ^2^ = 83.2, df = 1, p < 0.001), and an interactive effect between exposure time and temperature (χ^2^ = 55.1, df = 1, p < 0.001; **Fig 4**). Natural prevalence of viral infection was not evenly distributed enough among our treatment groups to adequately evaluate interactions between viral infection, temperature, and time, but we did identify a significant effect of the presence of DWV-B in untreated (time = 0) samples, which was associated with higher sperm viability (F = 5.9; df = 1, 49; p = 0.019; **S2 Fig in S1 File**). ABPV, IAPV, CBPV, and LSV were not detected in any samples, and while BQCV and DWV-A were detected, we found no association between the presence of these viruses and sperm viability (virus quantification data is available in **S1 Data**). The 60°C temperature was almost intolerable for sperm, with nearly all sperm dying within a 30 minute exposure.

### Experiment 5 –Virus inoculation reduces sperm heat resilience

**Fig 5:**
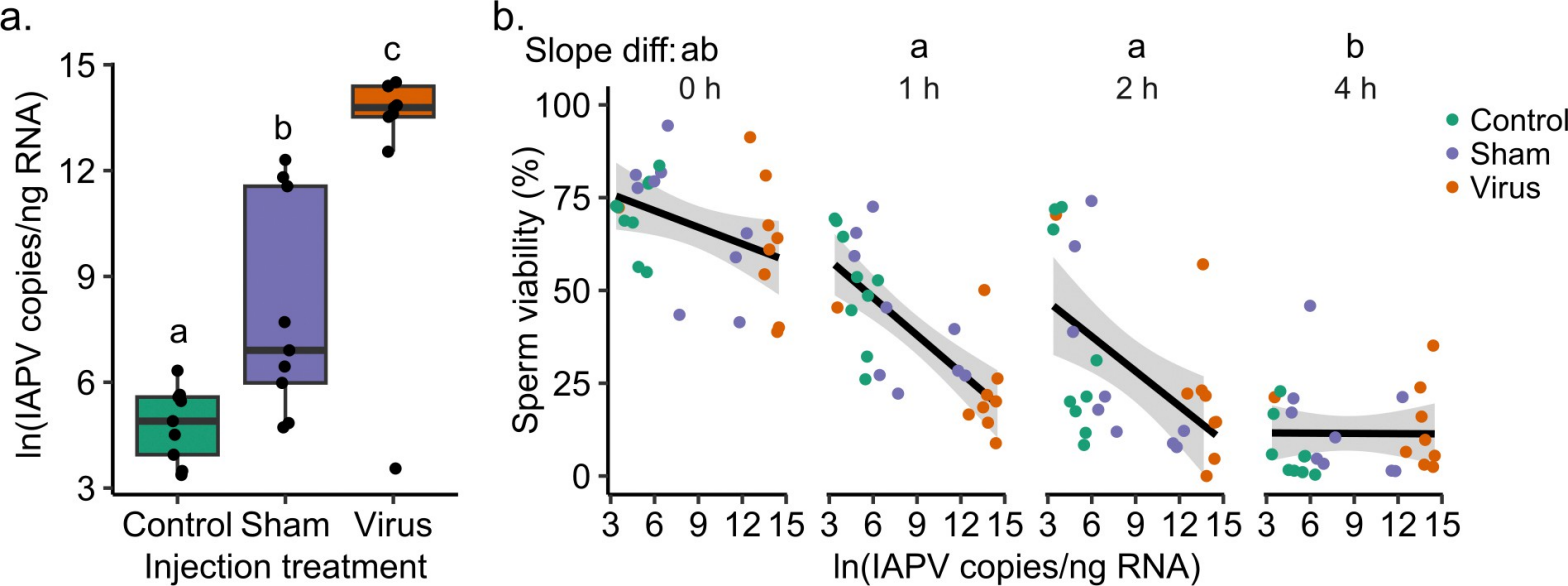
IAPV infection reduces sperm viability. We extracted semen from control, sham-inoculated (saline injection), and IAPV-inoculated (500 copies of infectious IAPV) drones (n = 9 each) three days after treatment, and exposed sperm to heat (52.5°C) for 0, 1, 2, or 4 hours. A) IAPV copies were determined by absolute qPCR quantification in the three experimental groups (control, sham, and virus). Boxes represent the interquartile range and whiskers span 1.5 times the interquartile range. Bars represent the median. Lowercase letters indicate significant differences between groups (p < 0.05, Tukey method of p-value adjustment). B) IAPV (χ^2^ = 15.6, df = 1, p < 0.001), time (χ^2^ = 310, df = 3, p < 0.001), and their interaction (χ^2^ = 22.3, df = 3, p < 0.001) all significantly and negatively predict sperm viability. Differences in slopes between time points are indicated with lower case letters (p < 0.05, Tukey method of p-value adjustment).

To test if virus inoculation affects sperm heat resilience, we assessed sperm viability of control, sham-inoculated, and IAPV-infected drones (n = 9 each) in response to heat (52.5°C) over time (0, 1, 2, and 4 h). We chose 52.5°C for this experiment because, while not a realistic exposure temperature for drone sperm, this temperature resulted in the steepest decline in sperm viability that could still be adequately modelled (**Fig 4**); therefore, we expected it to provide wide stratification in the data in which to see a potential protective effect of infection. We confirmed that IAPV copies varied significantly by group (F = 18.8; df = 2, 24; p < 0.001), with levels in the infected group being higher than the sham group by about three orders of magnitude (**Fig 5A**). However, we also observed higher infection levels in the sham group relative to the uninjected drones, which points to some level of viral transfer between groups, or possibly a general effect of wounding weakening natural immunity to existing infections. IAPV was thus best accounted for in our statistical model describing sperm viability as a continuous rather than categorical variable. Applying this approach, we found that, contrary to our predictions, IAPV (χ^2^ = 15.6, df = 1, p < 0.001) and time (χ^2^ = 310, df = 3, p < 0.001) significantly and negatively affect sperm viability. These variables also significantly interact (χ^2^ = 22.3, df = 3, p < 0.001), with the steepest decline of sperm viability with increasing IAPV occurring in the 1 h and 2 h heat-treatment time points (**Fig 5B; Table 2**).

**Table 2:** Post-hoc testing of sperm viability trends with IAPV intensity among time points.

Contrast	Estimate	T ratio	P value*
0 h– 1 h	0.021	2.2	0.13
0 h– 2 h	0.022	2.4	0.095
0 h– 4 h	-0.015	-1.7	0.35
1 h– 2 h	0.0012	0.13	1.0
1 h– 4 h	-0.036	-3.9	< 0.001
2 h– 4 h	-0.038	-4.0	< 0.001

*Tukey p-value adjustment method

## Discussion

In an effort to understand what traits contribute to heat resilience of honey bee drones and their mating potential (sperm viability), we investigated how population origin, body mass, and virus infection status influenced drone and sperm heat resilience in a series of experiments. Our main findings were that: 1) heavier drones were more likely to survive an acute heat challenge (**Fig 1**); 2) sperm from drones with different population origins within British Columbia had highly variable levels of heat resilience (**Fig 2**); 3) drones from Southern California were more likely to survive a heat challenge than drones originating from Northern California (**Fig 3**); 4) sperm from heavier drones were not more heat tolerant than sperm from smaller drones (**Fig 4**); and 5) adult-acquired viral infections reduced sperm heat resilience (**Fig 5**).

In our drone survival experiments, we tested a temperature and time frame (4 h at 42°C) that is in the realm of what drones might encounter inside or outside their hives during extreme conditions, though specific data on drone experiences in the field during heat waves is lacking. Our *in vitro* sperm challenge using BC drones used the same temperature and time frame. In the *in vitro* temperature-by-time array and the virus challenge experiments, however, the temperatures used may not be biologically relevant (and some, such as the 60°C treatment, are unlikely to occur in nature). Rather, the goal with these experiments was in part to define the boundaries within which sperm may still persist and to understand the sperm responses under the most extreme scenarios. Future endeavors should focus on better defining the range of possible drone experiences in the field, including internal body temperatures, and using these data to guide the conditions tested in the laboratory.

The finding that heavier drones were more likely to survive heat challenges is consistent with the positive relationship between body size and heat resilience that has been reported in some other insects [27–31], including males of another bee species, *Xenoglossa pruinosa* [32]. However, several previous studies also found no association between body size (or mass) and heat resilience (critical thermal maxima, or CT_max_) within or among other insect species [34, 35, 70–72]. The apparent species-specific nature of how body size relates to CT_max_ among insects suggests that other factors are also important determinants of heat resilience. It is possible that heating time rather than cooling capacity may be more influential for heat resilience during short-term exposures (as larger individuals take longer to overheat) compared to long-term exposures, but this does not explain the lack of relationship between CT_max_ and body size observed in many species (see above citations). Notably, in our experiments we recorded wet body mass, which is influenced by hydration state, whereas body size is not. Hydration can influence heat resilience (*e*.*g*., [28]) and is an important factor to account for because more hydrated individuals have both a higher specific heat capacity and greater ability to self-cool through evaporation [73]. Since larger-bodied individuals have a smaller surface area-to-volume ratio, and thus a more limited ability to self-cool, interactions between hydration state and body size would be enlightening to assess in our study system, and such an interaction might explain some of the inconsistencies in temperature-body size relationships observed in other species. We note that while it is possible that in our experiments some water evaporated from drones between the time of death and when they were weighed, thus reducing their body mass independent of their actual heat resilience, this effect was likely negligible as the majority of drone deaths occurred close to the 4 h exposure limit, at which time the experiment was terminated and all drones were weighed.

We predicted that drones from Southern California would be more likely to survive heat challenges than drones from Northern California, and this prediction was borne out (**Fig 3**). This is noteworthy because colonies in Southern California tend to have a greater proportion of African ancestry than the drones originating from Northern California (which is outside the hybrid zone) [55], and honey bees in Africa are likely well-adapted to thrive in hot conditions. We speculate that, due to the hotter climate in Southern California, these colonies are similarly under greater selection for heat resilience, in addition to having a history of selection in hot conditions before arriving in the U.S. This may be what led the Southern Californian colonies (which, in this experiment, had either feral origins or were breeding with the local southern population for multiple generations) to produce more heat resilient drones. However, the higher heat resilience of Southern Californian drones appears to be resulting from both a modest increase in heat tolerance as well as a surprisingly low survival of drones in the control group (**Fig 3**). Larger sample sizes in the control group and post-heat assessments of comorbidities in all groups (such as pathogenic infections) may help identify the underlying causes of this pattern in the future. Unfortunately, we do not have corresponding sperm viability data for these groups that are comparable to the sperm viability data presented throughout this body of work, nor did we obtain sperm viability data among drones from different genetic origins that survived experimental heat challenges, but this is an area of investigation that we are actively pursuing.

We did, however, compare sperm heat resilience among drones from alternate sources, and the sperm viability data from drones with diverse population origins within British Columbia showed remarkable variation in their response to heat (**Fig 2B**), prompting us to investigate other potential explanatory variables that may underly such extreme differences. While drone sampling by different BC beekeepers may have led to subjective assessments of age and possible differences in heat tolerance, we note that the variation in sperm response to heat seen in the UBC colony data (**Fig 2A** and **S1 Fig in S1 File)**, where drone age is controlled, is similar in spread to those from sources elsewhere in BC. We were interested in following this up by investigating interactive effects of viral infection, in particular, because of three key observations. First, we observed here that drones with naturally acquired DWV-B infections had, surprisingly, higher sperm viability than drones without DWV-B infection (**S2 Fig in S1 File**). Second, our previous research identified a positive relationship between at least one HSP and stored sperm viability [74]. Third, McMenamin *et al*. [41] have shown that heat stress has an antiviral effect; therefore, we hypothesized that a potential mechanism explaining these collective observations could lie in the dual role of HSPs as both antiviral and abiotic stress-mitigating agents. Although one might expect a viral infection to impair fitness of the individual, we initially reasoned that perhaps the elevation of HSPs stimulated by the virus could protect sperm from subsequent abiotic stress. However, when we tested this hypothesis by infecting adult drones, then subjecting their sperm to heat stress, we found the opposite—sperm from infected drones were less resilient to heat than sperm from control drones (**Fig 5B**), suggesting, sensibly, that there is a trade-off between sperm maintenance and immunity or perhaps a direct effect of pathogenic infection on reproductive quality. We have yet to reconcile this observation with the positive correlation between infection and sperm viability we found, but it is possible that the disagreement is due to the duration of infection before sperm sampling, pathogenicity of the virus (IAPV vs. DWV-B), life stage at which the drone became infected (infections acquired before, during, or after spermatogenesis may produce different outcomes), or a potential consequence of cellular resource limitation in drones that we have yet to deconvolute. Overall, these data support the idea that the negative effect of heat on drone sperm viability is exacerbated by viral infection ― a concerning finding, given that drones are also preferentially parasitized by the *Varroa destructor* mite, which is a potent virus vector [50].

The relatively small negative impact of adult-acquired viral infections we identified here (**Table 2**) could potentially explain only a small fraction of the variation in sperm heat resilience we found among drones originating within British Columbia. There are clearly additional influential factors when it comes to drone sperm heat resilience. An intriguing explanation might be genotype, but since we did not conduct genetic testing, we cannot separate environmental or management differences among operations from those potentially due to genetics. Research on *Drosophila* fruit flies has shown that male upper thermal fertility limits are better predictors of species distributions than CT_max_, so this trait is clearly under strong selective pressure among other species [75]. While upper thermal tolerance limit of sperm seems like a trait that should be evolutionarily constrained within a species, the broad global distribution of honey bees suggests that it might be more plastic in honey bees than other insects. It is also possible that the trait is protected from selection by the somewhat thermoregulated colony environment; a scenario which could also lead to great variation among individuals. Determining the factors underlying these male honey bee fertility differences should continue to be a priority as we begin to grapple with keeping honey bees in regions which were not historically at risk of extreme heat.

## Supporting information

S1 Data Drone survival and sperm viability raw data. All data underlying the figures and analyses conducted in this manuscript are provided in this file.
(XLSX)

S1 File Supplementary figures and tables. Contains S1 and S2 Figs as well as S1-S5 Tables.
(DOCX)
