# Supplementary material for: Factors affecting heat resilience of drone honey bees (Apis mellifera) and their sperm
Source: PLoS One. 2025 Feb 7;20(2):e0317672. doi: 10.1371/journal.pone.0317672 (PMC11805398; doi:10.1371/journal.pone.0317672)
Supplement: S1 File — Contains S1 and S2 Figs as well as S1-S5 Tables. (DOCX) [file pone.0317672.s002.docx]

Factors affecting heat resilience of drone honey bees (*Apis mellifera*) and their sperm

Alison McAfee^1,2,¶^*, Bradley N. Metz^2,¶^, Patrick Connor^2^, Keana Du^2^, Christopher W. Allen^3^, Luis A. Frausto^3,4^, Mark P. Swenson^3,4^, Kylah S Phillips^3,5^, Madison Julien^3^, Zoe Rempel^6^, Robert W. Currie^6^, Boris Baer^3,&^, David R. Tarpy^2, &^, and Leonard J. Foster^1, &,^*

1. Department of Biochemistry and Molecular Biology, Michael Smith Laboratories, University of British Columbia, Vancouver, British Columbia, Canada
2. Department of Applied Ecology, North Carolina State University, Raleigh, North Carolina, USA
3. Center for Integrative Bee Research (CIBER), Department of Entomology, University of California Riverside, Riverside, California, United States
4. Department of Microbiology & Plant Pathology, University of California Riverside, Riverside, California, USA
5. Department of Molecular, Cell & Systems Biology, University of California Riverside, Riverside, California, USA
6. Department of Entomology, University of Manitoba, Winnipeg, Manitoba, Canada

^¶^These authors contributed equally to this work

^&^These authors also contributed equally to this work

*Corresponding authors:

E-mail: [alison.n.mcafee@gmail.com](mailto:alison.n.mcafee@gmail.com) (AM)

Email: [foster@msl.ubc.ca](mailto:foster@msl.ubc.ca) (LJF)

**
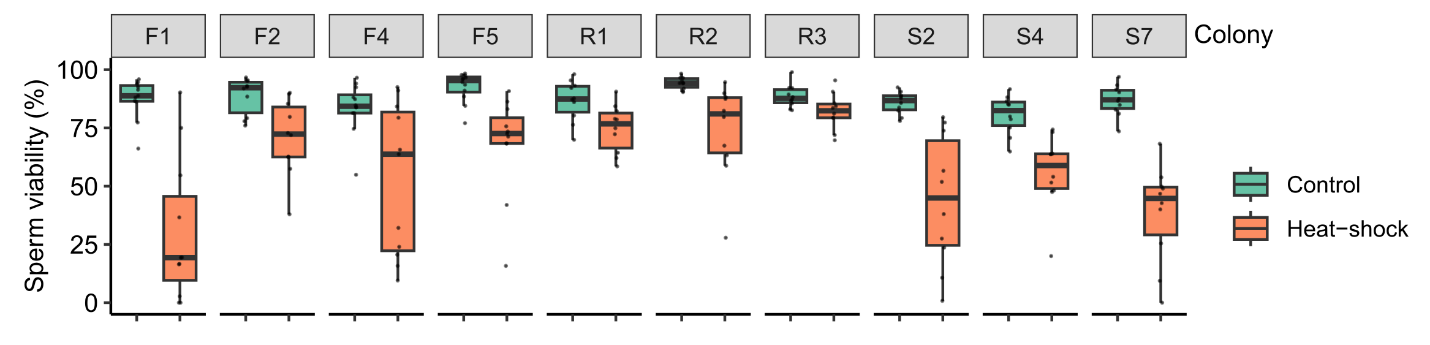
**

**S1 Figure. Colony-wise viability of *in vitro* sperm treatments.** These data are the same as Figure 2a, but plotted by colony.

**
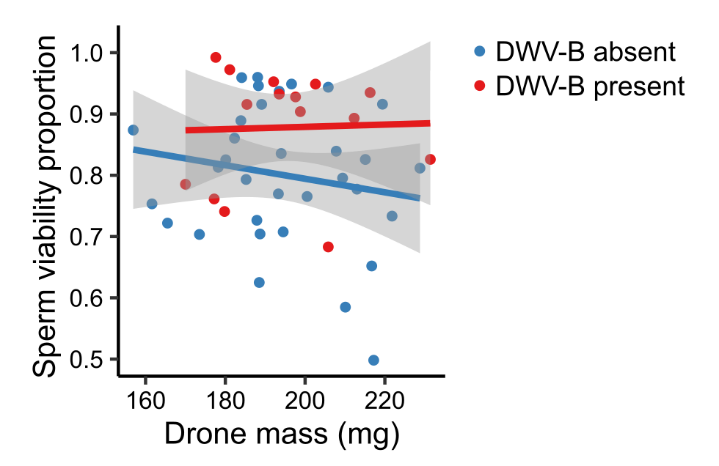
**

**S2 Figure. Relationship between DWV-B presence and sperm viability.** Among untreated semen samples (time = 0), there was no significant relationship between drone mass and sperm viability (F = 0.65, df = 1, p = 0.43), but presence of underlying DWV-B infections was associated with higher sperm viability (F = 5.97, df = 1, p = 0.019) (linear model of arcsine square root transformed sperm viability against drone mass (continuous), and DWV-B (categorical, levels: presence, absence)).

**S1 Table. Sample sizes for Experiment 1 survival challenge**

| Colony | Drone treatment group | Temperature (°C) | Drones - total | Drones - weighed* |
| --- | --- | --- | --- | --- |
| F1 | Heat | 42 | 50 | 35 |
| F1 | Control | 33 | 50 | - |
| F4 | Heat | 42 | 50 | 50 |
| F4 | Control | 33 | 50 | - |
| F2 | Heat | 42 | 50 | 50 |
| F2 | Control | 33 | 50 | - |
| R1 | Heat | 42 | 40 | 40 |
| R1 | Control | 33 | 20 | - |
| R3 | Heat | 42 | 40 | 38 |
| R3 | Control | 33 | 30 | - |
| R2 | Heat | 42 | 48 | 44 |
| R2 | Control | 33 | 20 | - |

*Only drones receiving the heat treatment were used to identify relationships between mass and survival. All dead drones were weighed but the high survival rates obtained (see Figure 1) meant it was not necessary to weigh every surviving drone.

**S2 Table. Sample sizes for Experiment 2 sperm viability challenges**

| Origin | Source | Colony | Sperm treatment group | Temperature (°C) | N |
| --- | --- | --- | --- | --- | --- |
| UBC | Australia | F1 | Heat | 42 | 11 |
|  | Australia | F1 | Control | 33 | 11 |
|  | Australia | F4 | Heat | 42 | 11 |
|  | Australia | F4 | Control | 33 | 11 |
|  | Australia | F5 | Heat | 42 | 11 |
|  | Australia | F5 | Control | 33 | 11 |
|  | Ukraine | F2 | Heat | 42 | 10 |
|  | Ukraine | F2 | Control | 33 | 10 |
|  | Ukraine | R1 | Heat | 42 | 10 |
|  | Ukraine | R1 | Control | 33 | 10 |
|  | Northern California | S7 | Heat | 42 | 10 |
|  | Northern California | S7 | Control | 33 | 10 |
|  | Northern California | S4 | Heat | 42 | 10 |
|  | Northern California | S4 | Control | 33 | 10 |
|  | Northern California | S2 | Heat | 42 | 10 |
|  | Northern California | S2 | Control | 33 | 10 |
|  | Northern California | R3 | Heat | 42 | 10 |
|  | Northern California | R3 | Control | 33 | 10 |
|  | Northern California | R2 | Heat | 42 | 10 |
|  | Northern California | R2 | Control | 33 | 10 |
| BC donors | Sunshine Coast | A | Heat | 42 | 10 |
|  | Sunshine Coast | A | Control | 33 | 10 |
|  | Fraser Valley | B | Heat | 42 | 10 |
|  | Fraser Valley | B | Control | 33 | 10 |
|  | North Okanagan | C | Heat | 42 | 7 |
|  | North Okanagan | C | Control | 33 | 8 |
|  | Central Okanagan | D | Heat | 42 | 9 |
|  | Central Okanagan | D | Control | 33 | 8 |
|  | South Okanagan | E | Heat | 42 | 7 |
|  | South Okanagan | E | Control | 33 | 9 |
|  | Nechako | F | Heat | 42 | 9 |
|  | Nechako | F | Control | 33 | 10 |

**S3 Table. Experiment 3 – Northern Californian vs. Southern Californian drone heat survival**

| Origin | Source | Colony | Drone treatment group | Temperature (°C) | N - total |
| --- | --- | --- | --- | --- | --- |
| Northern California | Package | 402 | Heat | 42 | 90 |
| Northern California | Package | 402 | Control | 35 | 30 |
| Northern California | Package | 408 | Heat | 42 | 116 |
| Northern California | Package | 408 | Control | 35 | 23 |
| Northern California | Nucleus colony | R01 | Heat | 42 | 120 |
| Northern California | Nucleus colony | R01 | Control | 35 | 50 |
| Southern California | Swarm | DHS1000 | Heat | 42 | 30 |
| Southern California | Swarm | DHS1000 | Control | 35 | 28 |
| Southern California | Swarm | R1001 | Heat | 42 | 90 |
| Southern California | Swarm | R1001 | Control | 35 | 30 |
| Southern California | Swarm | RT1002 | Heat | 42 | 25 |
| Southern California | Swarm | RT1002 | Control | 35 | 20 |
| Southern California | Multi-generation requeening | 3282 | Heat | 42 | 114 |
| Southern California | Multi-generation requeening | 3282 | Control | 35 | 28 |
| Southern California | Multi-generation requeening | 4240 | Heat | 42 | 118 |
| Southern California | Multi-generation requeening | 4240 | Control | 35 | 30 |

**S4 Table. Sample sizes for Experiment 4 sperm viability heat challenge**

| Colony* | Drones | Temperature (°C) | Time (min) | Repeated samples |
| --- | --- | --- | --- | --- |
| 25 | 8 | 30 | 0 | 8 |
|  |  |  | 35 | 8 |
|  |  |  | 65 | 8 |
|  |  |  | 125 | 8 |
|  |  |  | 245 | 8 |
| 423 | 1 | 45 | 0 | 1 |
|  |  |  | 35 | 1 |
|  |  |  | 65 | 1 |
|  |  |  | 125 | 1 |
|  |  |  | 245 | 1 |
| 424 | 7 | 45 | 0 | 7 |
|  |  |  | 35 | 7 |
|  |  |  | 65 | 7 |
|  |  |  | 125 | 7 |
|  |  |  | 245 | 7 |
| Blue-8 | 8 | 52.5 | 0 | 8 |
|  |  |  | 35 | 8 |
|  |  |  | 65 | 8 |
|  |  |  | 125 | 8 |
|  |  |  | 245 | 8 |
| 13 | 8 | 60 | 0 | 8 |
|  |  |  | 35 | 8 |
|  |  |  | 65 | 8 |
|  |  |  | 125 | 8 |
|  |  |  | 245 | 8 |

*Because colony confounds with treatment temperature in this experiment, we are unable to delineate temperature effects from colony effects, but given the extreme nature of the chosen temperatures, we expect temperature to be the dominant factor.

**S5 Table. Sample sizes for Experiment 5 sperm viability heat challenge (all at 52.5°C)**

| Round | Drones | Group | Time (min) | Repeated samples |
| --- | --- | --- | --- | --- |
| 1 | 6 | IAPV-inoculated | 0 | 6 |
|  |  |  | 60 | 6 |
|  |  |  | 120 | 6 |
|  |  |  | 240 | 6 |
| 2 | 3 |  | 0 | 3 |
|  |  |  | 60 | 3 |
|  |  |  | 120 | 3 |
|  |  |  | 240 | 3 |
| 1 | 6 | Sham-inoculated | 0 | 6 |
|  |  |  | 60 | 6 |
|  |  |  | 120 | 6 |
|  |  |  | 240 | 6 |
| 2 | 3 |  | 0 | 3 |
|  |  |  | 60 | 3 |
|  |  |  | 120 | 3 |
|  |  |  | 240 | 3 |
| 1 | 6 | Uninjected | 0 | 6 |
|  |  |  | 60 | 6 |
|  |  |  | 120 | 6 |
|  |  |  | 240 | 6 |
| 2 | 3 |  | 0 | 3 |
|  |  |  | 60 | 3 |
|  |  |  | 120 | 3 |
|  |  |  | 240 | 3 |
